# Matched serum- and urine-derived biomarkers of interstitial cystitis/bladder pain syndrome

**DOI:** 10.1371/journal.pone.0309815

**Published:** 2024-12-31

**Authors:** Tadeja Kuret, Igor Sterle, Rok Romih, Peter Veranič

**Affiliations:** 1 Institute of Cell Biology, Faculty of Medicine, University of Ljubljana, Ljubljana, Slovenia; 2 Department of Urology, University Medical Centre Ljubljana, Ljubljana, Slovenia; China Medical University, TAIWAN

## Abstract

Setting up the correct diagnosis of interstitial cystitis/bladder pain syndrome (IC/BPS), a chronic inflammatory disease of the bladder, is a challenge, as there are neither diagnostic criteria nor reliable and non-invasive disease biomarkers available. The aim of the present study was to simultaneously determine matched serum- and urine-derived biomarkers of IC/BPS, which would provide additional insights into disease mechanisms and set the basis for further biomarker validation. Our study included 12 female patients with IC/BPS and 12 healthy controls. A total of 33 different biomarkers were measured, including cytokines and chemokines, proteins involved in extracellular matrix remodeling, adhesion molecules, growth factors, and markers of oxidative stress using enzyme linked immunoassays and multiplex technology. Heatmaps and principal component analysis based on significantly altered biomarkers, revealed urine- and serum-associated IC/BPS signatures that clearly differentiated IC/BPS patients from controls. Four biomarkers, including CCL11, BAFF, HGF and MMP9, were significantly upregulated in both serum and urine of patients with IC/BPS compared to controls. Serum levels of MMP9 were associated with disease severity and could distinguish well between IC/BPS patients with and without Hunner’s lesions. Systemic levels of MMP9 can therefore mirror the local pathology within the bladders of IC/BPS patients, and MMP9 may prove to be a useful target for the development of novel therapeutic interventions. Utilizing a comprehensive panel of both urine and serum biomarkers, identified here, holds promise for disease detection in IC/BPS patients.

## Introduction

Interstitial cystitis/bladder pain syndrome (IC/BPS) is a chronic and debilitating inflammatory disease of the urinary bladder. It is generally defined as chronic pelvic pain lasting longer than six months, pressure or discomfort associated with bladder filling, accompanied by at least one other urinary tract symptom, such as frequency of urination, urinary urgency and nocturia, without evidence of a urinary tract infection or other identifiable pathological cause [1]. IC/BPS is more common in females than in males, with an estimated prevalence of 300 per 100,000 females [2, 3]. The male prevalence is considered to be 10–20% of the female estimate [4]. However, due to the complexity of the disease, the presence of non-specific clinical symptoms, several different clinical phenotypes and the lack of standardized diagnostic criteria or reliable diagnostic biomarkers, IC/BPS is likely to be underdiagnosed [5, 6]. The diagnosis of IC/BPS is currently mostly based on the exclusion of other diseases and conditions and varies considerably between different clinical centers worldwide [7]. There is an unmet need to identify novel candidate disease biomarkers in serum or urine samples that are obtained by a minimally invasive procedure for diagnostic purposes and to provide insights into disease mechanisms.

Depending on the pathological features of the patient, as determined by cystoscopy and histologic findings from bladder biopsies, IC/BPS can be classified as Hunner type (ulcerative) IC/BPS (HIC/BPS) or non Hunner type (non-ulcerative) IC/BPS (NHIC/BPS). HIC/BPS is defined by the presence of Hunner’s lesions (i.e., mucosal lesions accompanied by abnormal capillary structures), urothelial denudation, inflammation, immune cell infiltrates, and edema, whereas NHIC/BPS without Hunner’s lesions has minimal histologic changes in the bladder [8, 9].

Although the exact etiology and pathophysiology of IC/BPS remain unknown, there is a general agreement that the disease is multifaceted and has both local and systemic triggers [10]. Urothelial cell damage and dysfunction and activation of the immune response are considered to be key events contributing to a persistent inflammatory process that characterizes IC/BPS [11, 12]. More recent studies have shown that oxidative stress, resulting from either increased production and accumulation of reactive oxygen species (ROS) or insufficient capacity of the antioxidant defense system [13] is implicated in the pathogenesis of the disease [10, 14]. Several studies have already investigated various inflammatory cytokines and chemokines in urine as potential biomarkers for IC/BPS (a detailed review can be found in [15]), while only three studies have focused on oxidative stress biomarkers [16–18]. A limited number of studies reported the levels of other potential biomarkers besides cytokines and chemokines that play an important role in chronic inflammation, such as factors involved in extracellular matrix (ECM) remodeling, adhesion and angiogenesis [19, 20]. None of the previous studies in this field have measured the same biomarkers simultaneously in urine and serum of IC/BPS patients to investigate both local and systemic pathological processes.

The overall aim of the present study was to identify serum and urine-derived biomarkers of IC/BPS that would provide additional insight into disease mechanisms and set the basis for further biomarker validation leading to improved diagnostic measures in IC/BPS. We measured cytokines and chemokines, growth factors (GF), proteins involved in ECM remodeling, tissue repair and angiogenesis, adhesion molecules and markers of oxidative stress simultaneously in serum and urine of patients with IC/BPS, and determined urine- and serum-associated signatures that clearly separate IC/BPS patients from healthy controls. We also discovered that serum levels of MMP9 correlate well with the disease severity and can be used to differentiate Hunner from non Hunner type of IC/BPS.

## Methods

### Study subjects and clinical data collection

The study was conducted in accordance with the Declaration of Helsinki and was approved by the National Medical Ethics Committee of the Republic of Slovenia (approval no. 0120-496/2021/6). Each participant signed informed written consent to the use of their biological materials and clinical data. All samples were fully anonymized.

The current prospective study included 12 consecutive female patients diagnosed with IC/BPS who came for a follow-up visit at Department of Urology, University Medical Centre Ljubljana, Slovenia from 26.05.2022 to 21.11.2022. For comparison, 12 healthy female volunteers who had no history of urinary tract infection (UTI) and had no lower urinary tract symptoms, were included. Samples from healthy volunteers were obtained between 26.05.2022 and 21.11.2022.

IC/BPS diagnosis was based on the proposed guidelines of the European Society for the Study of Interstitial Cystitis (ESSIC), which include chronic pelvic pain, pressure, or discomfort perceived to be related to the urinary bladder accompanied by at least one other urinary symptom, such as persistent urge to void or increased urinary frequency, for more than 6 months and the exclusion of potentially similar diseases [7]. The workup included assessment of clinical symptoms, medical history, and urinalysis. Patients with active UTI were excluded from the study.

The assessment of clinical symptoms/disease severity was based on the Interstitial Cystitis Symptom Index (ICSI), also known as the O’Leary-Sant Symptom Index [21]. This standardized questionnaire is a widely used scale that assesses the 4 fundamental symptoms of IC/PBS, including bladder pain, urgency, frequency, and nocturia, by asking how often each is experienced. Each symptom is scored between 0 (none) and 6 (a lot). The ICSI index score is the sum of the item scores (ranging between 0 and 24) [22].

### Serum samples

Peripheral blood was collected from patients and controls into serum tubes, allowed to stand at room temperature for 30 min, and centrifuged (2000 ×g, 10 min, room temperature) to separate the serum, which was then aliquoted and stored at − 80°C, until subjected to further analysis.

### Urine samples

The second morning void urine sample was obtained from patients and controls at approximately the same time and collected under standard clinical/medical conditions. Urinalysis was simultaneously performed to confirm an infection-free status. Urine was immediately placed on ice and transferred to the laboratory for preparation. The urine volume was determined before the samples were centrifuged at 2000xg for 10 min at 4°C. The supernatant was separated into aliquots in 1.5 mL tubes (1 mL per tube) and preserved at -80°C. Before further analyses were performed, the thawed urine samples were centrifuged at 12,000xg for 15 min at 4°C, and the supernatants were used for subsequent measurements.

### Laboratory parameters

Among the routine laboratory parameters, we measured different markers of systemic inflammation, including leukocyte, thrombocyte counts, levels of hemoglobin in peripheral blood, and serum levels of C-reactive protein (CRP). To evaluate renal function, serum levels of urea, creatinine, and glomerular filtration rate were determined.

### Enzyme-linked immunoassays

Enzyme-linked immunoassays (ELISA) were performed in serum and urine samples using commercial ELISA kits in duplicates, according to manufacturer’s instructions. Absorbance at 450 nm with a reference wavelength set at 570 nm was measured on a microplate reader (Safire; Tecan, Mannedorf, Switzerland). The following ELISA kits were used in the present study: human IL6 ELISA MAX™ Deluxe Set, human IL8 ELISA MAX™ Deluxe Set, human CXCL10 ELISA MAX™ Deluxe Set (all Bio Legend, San Diego, CA, USA), human CXCL1 Duo Set Kit (R&D Systems, USA), human creatinine ELISA kit (Cayman Chemicals, Ann Arbor, MI, USA).

### Multiplex assay

We used human pre-mixed multi-analyte kits (Human Luminex Discovery Assay; R&D Systems; RD-LXSAHM-20 and RD-LXSAHM-05) according to the manufacturer’s instructions on automated immunoassay analyzer MagPix (Luminex xMAP Technology, Luminex Inc., Austin, TX, USA). The calibration curve for each molecule was analyzed with a five-parameter logistic curve (Milliplex Analyst, Merck). Serum and urine concentrations of four groups of proteins (25 in total) were determined in our study: 1) cytokines and chemokines, including C-C motif chemokine ligands CCL2, CCL5, CCL11, CCL21, CCL27, interleukins IL4, IL1RA, interferons IFNα, IFNγ, B-cell activating factor (BAFF), tumor necrosis factor (TNF)-related apoptosis-inducing ligand (TRAIL), 2) factors involved in ECM remodeling and repair, including matrix metalloproteinases MMP1, MMP2, MMP9, MMP13, chitinase 3 like protein 1 (CHI3L1), trefoil factor (TFF)3, 3) growth factors (GF), including heparin-binding elongation factor G-like GF (HB-EGF), hepatocyte GF (HGF), vascular endothelial GF (VEGF), nerve GF (NGF), and 4) adhesion molecules, including intracellular adhesion molecule 1 (ICAM1), vascular cell adhesion molecule 1 (VCAM1), soluble L-selectin (sCD62L), and N-cadherin.

### Oxidative stress assays

Commercial ELISA and Antioxidant assay kits, purchased from Cayman Chemicals, Ann Arbor, MI, USA, were used to determine several markers of oxidative stress in serum and urine samples in duplicates, according to manufacturer’s instructions. DNA/RNA oxidative damage was determined by measuring multiple oxidized guanine species, released into the urine, including 8-hydroxyguanosine (8-OHG), 8-hydroxy-2’-deoxyguanosine (8-OHdG), and 8-hydroxyguanine. Lipid peroxidation in urine and serum samples was determined by quantifying the levels of malondialdehyde (MDA) through a controlled reaction with thiobarbituric acid, generating thiobarbituric acid reactive substances (TBARS). 8-izoprostane was quantified in serum and urine samples and total antioxidant activity of serum and urine was determined using Antioxidant assay kit.

### Statistical analysis

Statistical analysis was performed using Graph Pad Prism software 8.01 (Graphpad Software Inc., San Diego, CA, USA) and IBM SPSS Statistics for Windows, v22 (IBM, Armonk, NY, USA). Heatmaps and principal component analysis were performed using a freely accessible web server SRplot [23]. The normality of data distribution was calculated by the Shapiro-Wilk test. Summary statistics are expressed as medians and 25th–75th percentiles (Q_25_–Q_75_). Statistical differences between two groups were calculated using Mann-Whitney U-test or unpaired t-test with or without Welch’s correction for continuous variables depending on the normality of data distribution. One-sample Wilcoxon signed rank test was used to compare the median concentrations of biomarkers only detected in one group (control was set to 0). For correlation analysis, Spearman’s rank correlation coefficient was calculated. Receiver operating characteristic curves (ROC) were generated to assess the ability of the target biomarker to distinguish between HIC/BPS and NHIC/BPS and the areas under the ROC curve (AUC) were calculated. All tests were two-tailed and p values of <0.05 were regarded as statistically significant.

## Results

### Clinical and laboratory characteristics of patients with IC/BPS

Our study enrolled twelve female patients diagnosed with IC/BPS with median (Q_25_-Q_75_) age of 68 (65–71) years, and twelve female healthy controls with median age of 58 (55–60). Clinical and laboratory characteristics of patients with IC/BPS and controls are shown in Table 1. There were 7 (58%) patients with the presence of Hunner’s lesions in the bladder, defined as regions of focal inflammation that were present upon cystoscopy and confirmed by histology, and the median (Q_25_–Q_75_) ICSI score was 15.5 (7–18.5). All patients had median laboratory values of leukocytes, thrombocytes, haemoglobin and CRP within normal reference ranges. The serum levels of urea and creatinine in all patients were also within normal reference ranges, while the median glomerular filtration rate was significantly (p = 0.0038) lower than 90 ml/min.

**Table 1:** Demographic, clinical and laboratory data of IC/BPS patients and controls.

	IC/BPS	HC
***Demographic data***		
Number of patients	12	12
Median age in years (Q_25_–Q_75_)	68 (65–71)	58 (55–69)***
***Clinical data***		
Hunner’s lessions n (%)	7 (58)	
Median ICSI score (Q_25_–Q_75_)	15.5 (7–18.5)	
***Median laboratory values (Q***_***25***_ ***–Q***_***75***_ ***)***		***Reference ranges in HC***
Leukocytes (10^9^/l)	5.1 (4.2–6.6)	4.0–10.0
Thrombocytes (10^9^/l)	219.5 (190.5–244.8)	150–410
Haemoglobin (g/l)	134.5 (124.3–140.5)	120–150
CRP (mg/l)	<5	<5
Urea (mmol/l)	5.5 (4.9–6.4)	2.5–7.2
Creatinine (μmol/l)	67.0 (60.3–75.5)	44.0–97.0
Glomerular filtration rate (ml/min)	79.0 (69.5–89.3)	>90

***p<0.001; IC/BPS, interstitial cystitis/bladder pain syndrome; HC, healthy controls; ICSI, interstitial cystitis symptom index; CRP, C-reactive protein.

### Urine-derived biomarkers of IC/BPS

Since urinary biomarkers are particularly attractive due to the direct contact of urine with the urothelial cells of the bladder and the ease of sample collection, we first determined the urinary levels of 29 different proteins belonging to four different groups (cytokines and chemokines, factors involved in ECM remodeling and repair, GF, adhesion molecules), as well as four different markers of oxidative stress, including oxidized guanine species, 8-isoprostane, MDA and total antioxidant activity. To correct the values of measured biomarkers for the different degrees of hydration and protein excretion in each urine sample [24, 25], we normalized the concentration values of the biomarkers to the concentration values of urine creatinine (normalized concentration: pg/μg) in each sample. The urinary levels of CCL27, IL6, VCAM1 and MDA were below the detection limit of the method used for both IC/BPS and controls and were therefore excluded from further analysis. Out of the remaining 26 proteins tested, 11 showed significant changes between the two groups (Table 1). Patients with IC/BPS had significantly higher median normalized concentrations of chemokines CCL5 (p = 0.0005), CCL11 (p = 0–0045), CXCL10 (p = 0.0007), cytokine IL4 (p = 0.0205), and BAFF (p = 0.0387) compared to controls. Patients with IC/BPS also had higher median normalized concentration of biomarkers involved in tissue repair, including MMP1 (p = 0.005), MMP2 (p = 0.0173), and MMP9 (p = 0.0205), growth factor HGF (p = 0.0205) and adhesion protein N-cadherin (p = 0.0024), as compared to median normalized concentrations determined in controls. Median normalized urinary levels of oxidized guanine species (p = 0.0100) and 8-izoprostane (p = 0.0449) were significantly higher in IC/BPS vs controls (Table 2). Two biomarkers, CCL5 and MMP1, could be detected in urine samples of all IC/BPS patients, but in none of the samples from controls with the method used.

**Table 2:** Normalized concentrations of biomarkers in urine samples of IC/BPS patients and controls.

Biomarker (pg/μg)	IC/BPS (n = 12)		HC (n = 12)		P value	Sign.
	Median	Q_25_-Q_75_	Median	Q_25_-Q_75_		
**Cytokines and chemokines**						
CCL2	0.723	0.084–1.842	0.206	0.093–0.894	0.3859	ns
**CCL5**	**0.315**	**0.181–0.480**	**Below limit of detection**	**0.0005**	***
**CCL11**	**0.663**	**0.394–1.501**	**0.176**	**0.121–0.288**	**0.0045**	**
CCL21	0.177	0.109–0.465	0.155	0.113–0.279	0.5512	ns
CXCL1	0.229	0.131–0.551	0.173	0.072–0.378	0.4095	ns
**CXCL10**	**2.053**	**1.072–6.050**	**0.487**	**0.201–0.856**	**0.0007**	***
**IL4**	**0.200**	**0.182–0.323**	**0.168**	**0.105–0.192**	**0.0205**	*
IL8	0.079	0.000–0.202	0.230	0.155–0.287	0.0544	ns
IL1Rα	89.88	58.43–105.5	88.82	22.90–115.9	0.8959	ns
IFNα	0.034	0.027–0.044	0.032	0.027–0.053	0.7987	ns
IFNγ	0.163	0.110–0.309	0.158	0.115–0.237	0.5512	ns
**BAFF**	**0.095**	**0.058–0.188**	**0.043**	**0.014–0.078**	**0.0387**	*
TRAIL	0.041	0.024–0.201	0.051	0.037–0.099	0.6297	ns
**Growth factors**						
HBEGF	0.499	0.391–0.720	0.452	0.353–0.647	0.3186	ns
**HGF**	**0.675**	**0.191–0.775**	**0.241**	**0.201–0.288**	**0.0205**	*
NGF	0.013	0.011–0.017	0.011	0.010–0.015	0.4428	ns
VEGF	1.220	0.891–1.981	0.942	0.555–1.569	0.1600	ns
**Factors involved in ECM degradation and tissue repair**						
TFF3	59.17	51.96–62.30	55.07	48.41–75.56	0.9323	ns
**MMP1**	**0.288**	**0.124–1.20**	**Below limit of detection**	**0.0005**	***
**MMP2**	**10.50**	**5.28–18.73**	**3.31**	**1.00–9.14**	**0.0173**	*
**MMP9**	**112.4**	**31.73–145.8**	**9.77**	**0.774–88.12**	**0.0205**	*
MMP13	3.866	2.776–6.183	4.292	3.643–8.066	0.4428	ns
CHI3L1	8.92	5.23–13.85	7.72	5.47–13.09	0.8874	ns
**Cell adhesion proteins**						
**N-cadherin**	**144.4**	**27.75–295.4**	**30.31**	**22.48–43.48**	**0.0024**	**
ICAM1	362.1	46.24–1240	124.0	86.97–452.1	0.4428	ns
CD62L	59.87	33.52–94.17	45.00	28.24–60.79	0.3186	ns
**Oxidative stress markers**						
**Oxidized guanine**	**0.132**	**0.091–0.359**	**0.082**	**0.058–0.094**	**0.0100**	*
**8-izoprostane**	**9.101**	**5.165–12.80**	**3.222**	**1.263–8.543**	**0.0449**	*
Total antioxidant capacity (mM)	0.060	0.029–0.137	0.068	0.053–0.078	0.5137	ns

Urine levels of biomarkers were normalized to the urine concentration of creatinine in each sample (pg/μg creatinine). Significantly altered biomarkers are bold. *p<0.05

**p<0.01

***p<0.001; IC/BPS, interstitial cystitis/bladder pain syndrome; HC, healthy control; ECM, extracellular matrix.

To verify that the significant difference in urinary biomarkers was truly a consequence of the disease and not due to the significant difference in median age between IC/BPS and HC (68 vs. 58, p<0.0001), we correlated significantly altered urinary biomarkers with age in both groups. There was no correlation between selected biomarkers and age in IC/BPS patients (S1 Table), however, the normalized levels of MMP9 positively correlated with age in controls (Spearman’s r = 0.677, 95% CI from 0.150 to 0.905, p = 0.0182; S2 Table).

### Serum-derived biomarkers of IC/BPS

Several studies suggested that IC/BPS is a systemic inflammatory disease with a clear involvement of the immune system [12, 26, 27]. Since the classic laboratory markers of inflammation (CRP, leukocytes) were not elevated in IC/BPS patients (Table 1), we next sought to determine the levels of other serum biomarkers that might indicate systemic involvement in IC/BPS. We again determined the levels of 29 different proteins and three markers of oxidative stress, as described above for urine samples. Serum levels of CXCL10 and MDA were below the limit of detection of the method used and were excluded from further analysis. Out of the remaining 28 proteins, 11 were significantly different between the two groups. Median serum levels of chemokines CCL11 (p = 0.0005), CCL27 (p = 0.0018), cytokines IL1Rα (p = 0.0345), IFNα (0.0175) and BAFF (0.0127), growth factors HGF (p = 0.0025) and VEGF (p = 0.0151) were significantly higher, while median serum levels of HBEGF (p = 0.0088) were significantly lower in patients with IC/BPS vs HC. Serum levels of proteins involved in ECM degradation, including MMP9 (p = 0.0242) and CHI3L1 (p = 0.0284), involved in tissue repair, as well as adhesion molecule ICAM1 (p = 0.0014) were significantly higher in IC/BPS patients compared to controls. Median serum levels of 8-izoprostane (p = 0.0092) were significantly higher while the total antioxidant capacity (p = 0.0257) was significantly lower in IC/BPS patients as compared to controls (Table 3).

**Table 3:** Levels of measured biomarkers in serum samples of patients with IC/BPS vs controls.

Biomarker (pg/ml)	IC/BPS (n = 12)		HC (n = 12)		P value	Sign.
	Median	Q_25_-Q_75_	Median	Q_25_-Q_75_		
**Cytokines and chemokines**						
CCL2	282.2	210.3–410.7	221.1	177.9–304.3	0.1944	ns
CCL5 (ng/ml)	33.00	26.65–44.16	35.81	27.41–47.56	0.4600	ns
**CCL11**	260.3	201.8–317.6	169.4	109.8–201.9	**0.0005**	***
CCL21	379.1	366.0–526.3	359.4	315.9–405.2	0.1099	ns
**CCL27**	**529.4**	**459.2–590.0**	**386.8**	**337.7–429.0**	**0.0018**	**
CXCL1	23.24	17.73–43.94	29.71	19.60–35.67	0.7553	ns
IL4	104.6	101.3–128.3	107.8	94.64–117.2	0.5581	ns
IL6	18.27	5.67–34.72	29.74	8.91–77.89	0.4095	ns
IL8	15.30	13.33–75.41	0.234	0.063–16.41	0.0582	ns
**IL1Rα**	**649.4**	**484.0–807.4**	**484.5**	**346.0–654.2**	**0.0345**	*
**IFNα**	**6.78**	**5.63–7.53**	**5.17**	**4.57–6.28**	**0.0175**	*
IFNγ	127.8	98.29–149.3	118.7	102.1–127.8	0.5200	ns
**BAFF**	**792.8**	**696.2–1079**	**661.2**	**587.0–768.2**	**0.0127**	*
TRAIL	80.60	42.51–85.54	64.58	45.93–74.89	0.4854	ns
**Growth factors**						
**HBEGF**	**138.7**	**102.1–191.3**	**190.0**	**162.4–245.1**	**0.0088**	**
**HGF**	**215.8**	**173.6–263.9**	**136.5**	**129.8–163.0**	**0.0025**	**
NGF	5.94	3.84–6.66	4.53	3.20–5.14	0.2066	ns
**VEGF**	**124.5**	**76.98–153.4**	**68.36**	**54.11–83.41**	**0.0151**	*
**Factors involved in ECM and tissue repair**						
TFF3	2514	1911–3233	2166	1821–2587	0.4776	ns
MMP1	2692	965.1–8083	3728	2027–7558	0.6707	ns
MMP2 (ng/ml)	432.3	378.1–558.5	553.9	474.7–663.1	0.0984	ns
**MMP9 (ng/ml)**	**283.9**	**160.3–422.0**	**114.5**	**96.5–151.6**	**0.0242**	*
MMP13	698.7	555.9–1012	734.3	573.9–839.5	0.6128	ns
**CHI3L1 (ng/ml)**	**29.82**	**21.03–46.46**	**20.37**	**16.22–24.07**	**0.0284**	*
**Cell adhesion proteins**						
N-cadherin (ng/ml)	196.1	150.9–257.9	233.5	168.6–256.3	0.4507	ns
**ICAM1 (ng/ml)**	**410.9**	**295.9–560.3**	**243.5**	**126.5–301.4**	**0.0014**	**
VCAM1 (ng/ml)	653.1	606.8–818.6	645.6	366.0–760.3	0.3397	ns
CD62L (ng/ml)	842.8	735.3–876.6	934.1	894.2–985.1	0.1366	ns
**Oxidative stress markers**						
**8-izoprostane**	**83.29**	**63.72–105.3**	**27.75**	**21.40–79.34**	**0.0092**	**
**Total antioxidant capacity (mM)**	**4.48**	**3.86–4.79**	**5.19**	**4.45–5.68**	**0.0257**	*

Significantly altered biomarkers are bold.

*p<0.05

**p<0.01

***p<0.001; IC/BPS, interstitial cystitis/bladder pain syndrome; HC, healthy control; ECM, extracellular matrix.

Similar to the urine biomarkers, we also correlated significantly altered serum biomarkers with age in both groups to test whether the age difference between the groups influenced the significant differences. We observed no correlation between selected (statistically significantly changed) biomarkers and age in IC/BPS (S3 Table) or control group (S2 Table).

### IC/BPS-associated urine and serum signatures clearly separate IC/BPS patients from controls

**Fig 1:**
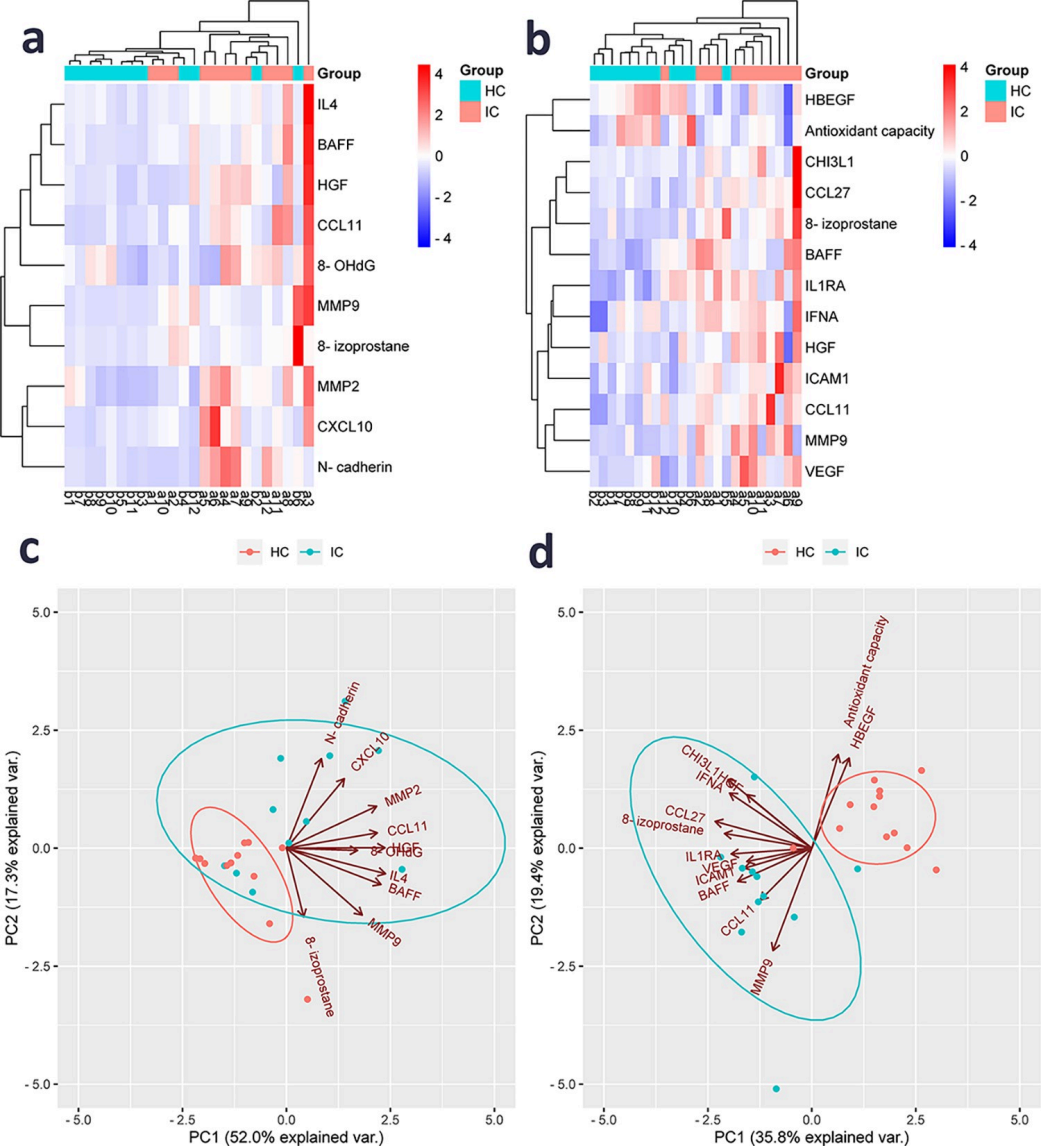
Heatmaps (a, b) and principal component analysis (c, d) for IC/BPS patients and HC in urine (a, c) and serum (b, d) based on significantly deregulated biomarkers. (a, b) Heatmaps with unsupervised hierarchical clustering based on significantly deregulated biomarkers in urine (a) and serum (b) showing a distinction between IC/BPS (pink; n = 12, a1-a12) and HC (blue; n = 12, b1-b12). Both rows and columns are clustered using Euclidean distance and complete linkage. (c, d) Principal component analysis based on significantly deregulated biomarkers in urine (c) and serum (d) showing a distinction between IC/BPS (blue; n = 12) and HC (pink; n = 12). Unit variance scaling was applied to rows and singular value decomposition (SVD) with imputation was used to calculate principal components. Prediction ellipses are such that with probability 0.68, a new observation from the same group will fall inside the ellipse. IC/BPS, interstitial cystitis/bladder pain syndrome; HC, healthy control; PC, principal component.

To further explore our biomarker dataset, we performed hierarchical clustering of subjects and significantly modulated urine and serum analytes. A moderately clear delineation between IC/BPS and HC based on significantly modified urine biomarkers was observed (three patients clustered into the HC group; Fig 1A), while the delineation based on serum biomarkers was far more clear (only one patient clustered into the HC group; Fig 1B), as reflected in the heatmaps. Finally, we investigated the variation patterns in the biomarker dataset using principal component analysis (PCA) and visualized principal components (PC) 1 and 2. For urine signature, PC1 and PC2 explain 52.0% and 17.3% (Fig 1C), while for serum signature, PC1 and PC2 explain 35.8% and 19.4% of the total variance (Fig 1D, respectively, with PC1 mainly contributing to the separation in both cases. The same patient (a12) clustered into the HC group in the heatmap, as well as in PCA showing a serum biomarker profile more similar to HC than to IC/BPS patients. This patient presented without Hunner’s lessions and had the lowest ICSI score (= 1) in our study.

### Levels of BAFF and CCL11 show a good positive correlation in urine and serum of IC/BPS patients

**Fig 2:**
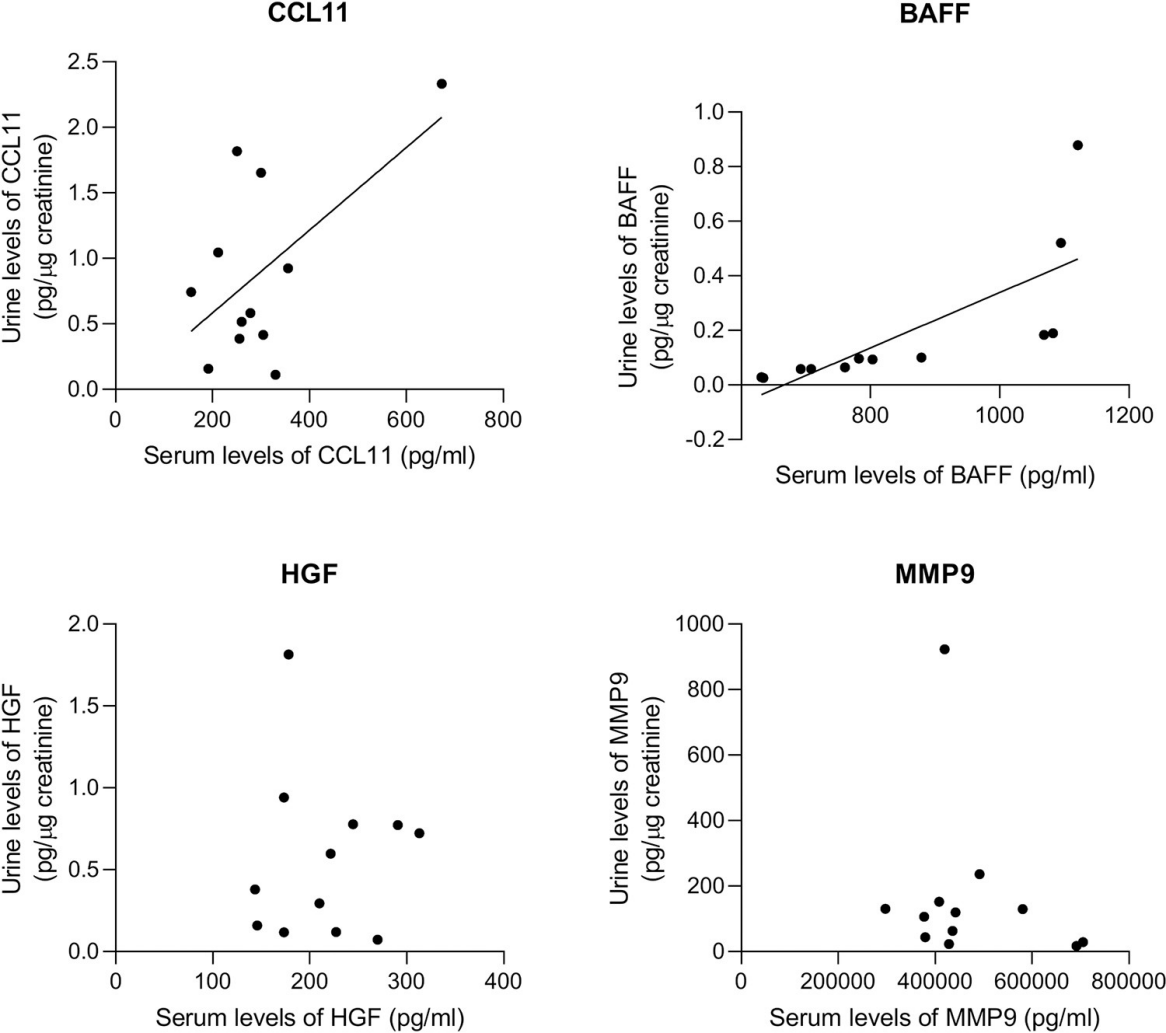
Correlation between serum and urine levels of selected biomarkers in IC/BPS patients. Shown is linear regression fitting line where applicable. IC/BPS, interstitial cystitis/bladder pain syndrome; CCL11 (eotaxin1), C-C motif chemokine 1; BAFF, B-cell activating factor; HGF, hepatocyte growth factor; MMP9, matrix metalloproteinase 9.

There were 4 biomarkers, namely CCL11, BAFF, HGF and MMP9, which were significantly elevated in both urine (3.77x, 2.21x, 2.8x, 11.5x, respectively) and serum (1.54x, 1.20x, 1.58x, 2.48x, respectively) samples from IC/BPS patients compared to controls. To determine whether the systemic levels of these biomarkers reflect the local levels in urine/bladder, we calculated correlation coefficients between them in IC/BPS patients. There was a good positive correlation between urine and serum levels of CCL11 (Spearman’s r = 0.591, 95% CI from 0.026 to 0.870, p = 0.0430) and BAFF (Spearman’s r = 0.750, 95% CI from 0.310 to 0.926, p = 0.0050), but not of HGF (Spearman’s r = 0.0155, 95% CI from -0.563 to 0.584, ns) and MMP9 (Spearman’s r = -0.209, 95% CI from -0.699 to 0.414, ns) (Fig 2). No correlation between any of the measured serum and urine biomarkers was observed in controls.

### Serum level of MMP9 is associated with disease severity

**Fig 3:**
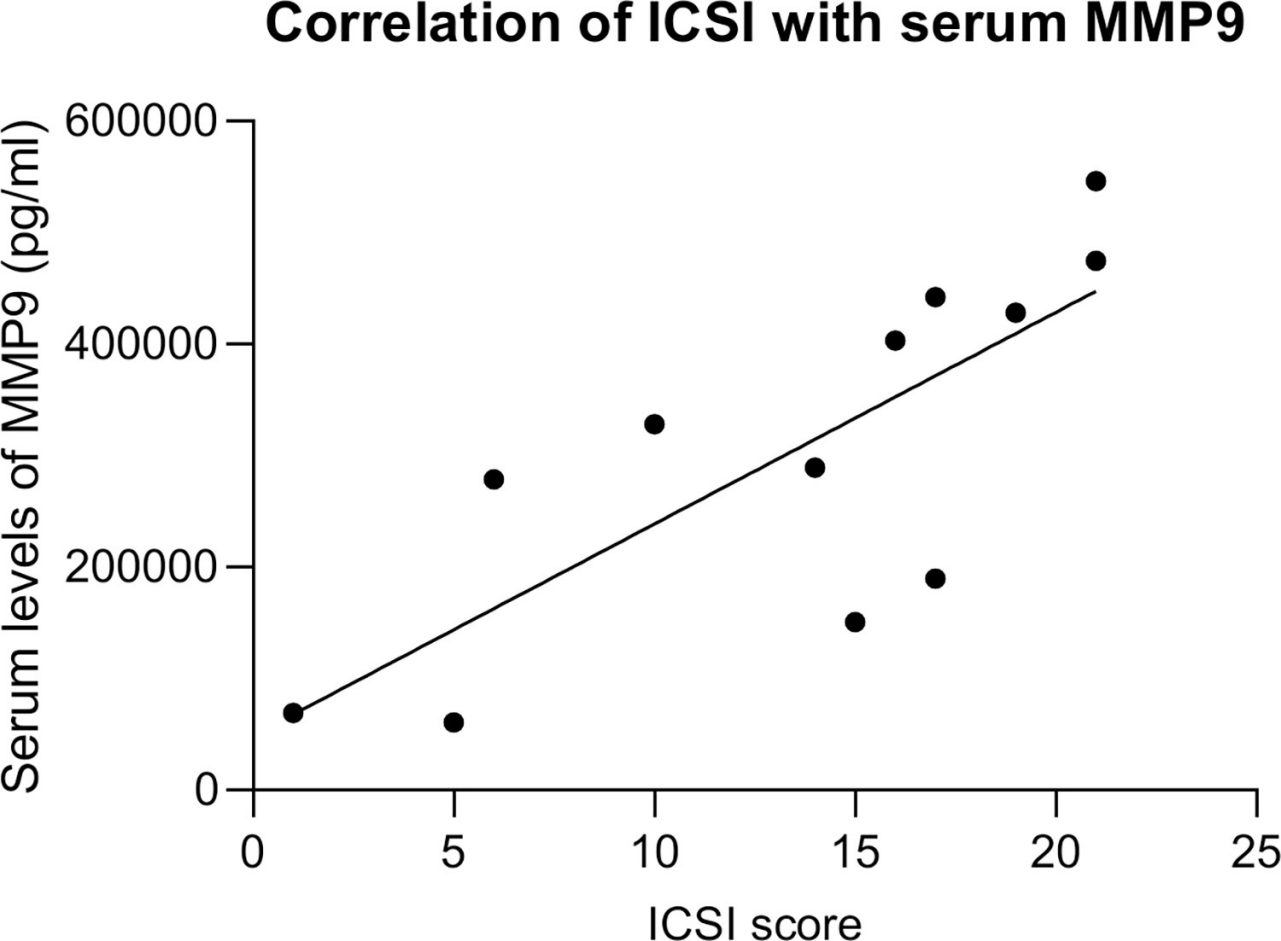
Correlation of ICSI score with serum MMP9 in IC/BPS patients. Shown is linear regression fitting line. IC/BPS, interstitial cystitis/bladder pain syndrome; ICSI, interstitial cystitis symptom index; MMP9, matrix metalloproteinase 9.

The standardized questionnaire ICSI used in our study to assess disease severity includes all critical parameters of IC/BS, such as urgency, daytime frequency, nocturnal frequency, and bladder pain. The ICSI is also a reliable and validated tool in the evaluation and management of IC/BPS patients, and regularly used in clinical trials of new therapies [28]. To investigate, whether there are biomarkers in our dataset associated with disease severity in IC/BPS patients, we correlated ICSI scores with levels of biomarkers, determined in serum and urine. There was a good positive correlation between ICSI score and serum levels of MMP9 (Spearman’s r = 0.825, 95% CI from 0.461 to 0.951; p = 0.0016) (Fig 3), but with none of the other measured biomarkers.

### Serum levels of MMP9 and ICSI score can separate HIC/BPS from NHIC/BPS

**Fig 4:**
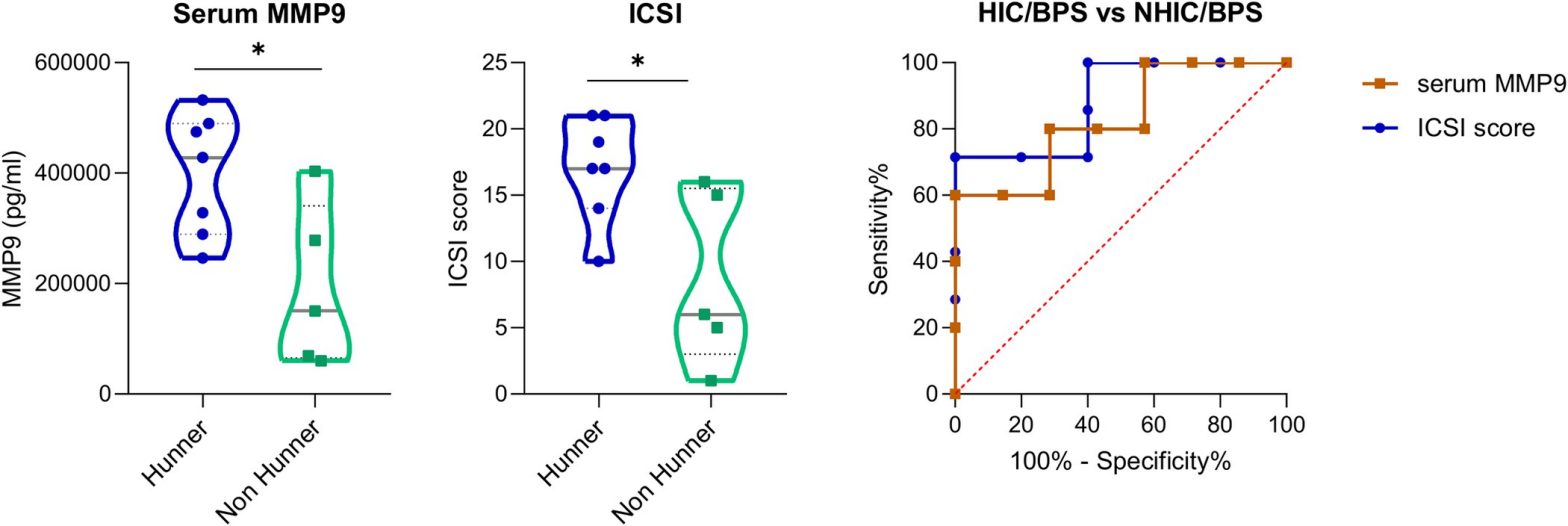
Differences in serum MMP and ICSI score between Hunner (HIC/BPS) and non Hunner (NHIC/BPS) type of IC/BPS. Shown are violin plots with median and Q_25_-Q_75_ for serum MMP (a), ICSI score (b) in HIC/BPS (blue) and NHIC/BPS (green) and ROC curves for serum MMP9 (blue circles) and ICSI score (orange squares) (c). *p<0.005; HIC/BPS, Hunner type of interstitial cystitis/bladder pain syndrome; NHIC/BPS, non Hunner type of interstitial cystitis/bladder pain syndrome; ICSI, interstitial cystitis symptom index; MMP9, matrix metalloproteinase 9.

Recent studies suggest that HIC/BPS, defined by the presence of Hunner’s lesions in the bladder, should be considered a distinct form of the disease and managed and treated accordingly [12, 29]. Currently, an invasive procedure (cystoscopy or biopsy) must be performed to categorise patients into HIC or NHIC/BPS, and we cannot distinguish between the two major forms of IC/BPS based on clinical symptoms or physical examination alone [29]. We have therefore aimed to identify biomarkers in urine or serum obtained by a less invasive technique that can distinguish between the two groups. After performing statistical analysis of all biomarkers, we discovered that patients with HIC/BPS (n = 7; 58%) had significantly higher median serum level of MMP9 (428.21 ng/ml (289.21–489.56) vs 150.58 ng/ml (65.02–340.93); p = 0.0303) compared to patients with NHIC/BPS (Fig 4A). No difference between HIC and NHIC/BPS patients was observed for any other measured biomarker. As expected, patients with Hunner’s lessions had more severe bladder symptoms reflected in higher median (Q_25_-Q_75_) ICSI score (17.0 (14.0–21.0) vs. 6.0 (3.0–15.5); p = 0.0265) compared to patients without Hunner’s lessions (Fig 4B). To evaluate the diagnostic value of serum MMP and ICSI to distinguish between HIC and NHIC/BPS, we performed ROC curve and calculated AUC. Both markers, MMP and ICSI score demonstrated high AUC (i.e., <0.7) with serum MMP9 reaching an AUC of 0.829 (p = 0.0618) and ICSI score reaching 0.886 (p = 0.0284*) with a cut-off value of 283.93 ng/ml for MMP9 (sensitivity of 80.00% and specificity of 71.43%) and a cut-off value of 15.5 for ICSI score (sensitivity of 71.43% and specificity of 80.00%) (Fig 4C).

## Discussion

Setting up the correct diagnosis of IC/BPS as early as possible is challenging with no diagnostic biomarkers available, disagreement in disease definition, several different clinical phenotypes and uncertainty about the pathophysiology and etiology of the disease [30]. There is evidence that less than 10% of patients presenting with clinical symptoms and signs characteristic of IC/BPS are formally diagnosed [5]. The delay from symptom onset to diagnosis is very common and is estimated to range from 3 to 7 years although early detection of patients with IC/BPS is crucial [30, 31]. Currently, diagnosis is mostly based on exclusion criteria and findings from cystoscopy or histologic examination of bladder biopsies [32, 33]. While cystoscopy and biopsy are effective tools for detecting Hunner type of IC/BPS, patients without Hunner’s lesions with minimal histologic changes in the bladder may still be misdiagnosed [8]. Moreover, both procedures are invasive and often have adverse effects such as infection, dysuria, frequency and hematuria [34, 35]. Not surprisingly, several studies have aimed to determine biomarkers for IC/BPS in urine [15, 18, 36–38], which is readily accessible, non-invasively collected and relatively easy to process, to support and improve the earlier diagnosis of IC/BPS [39, 40]. However, to date, no urine biomarker has found widespread use due to lack of reproducibility of results and insufficient evidence of clinical utility. This may be due in part to the complex composition of urine samples, inter-individual variability and the need for normalization, which is not yet a standardized procedure [41]. Another problem with using urine as a sample source is the risk of contamination during collection or handling, which may affect the accuracy of biomarker detection [42]. In contrast to urine samples, serum samples are subject to fewer challenges and limitations in terms of contamination during collection or handling, variability and the need for normalization [43].

Here, we included both urine and serum biomarkers in our study to investigate local and systemic involvement in IC/BPS. The elevated levels of inflammatory factors secreted from various types of cells within the bladder wall likely permeate into the systemic circulation and are excreted in the urine. On the other hand, it has been shown that systemic inflammation can lead to peripheral afferent nerve hyperexcitability, causing series of bladder symptoms such as urinary frequency and urgency [44]. In this case, higher levels of systemic inflammatory markers would be primarily due to systemic inflammation that triggers local symptoms. What remains unclear is whether a particular biomarker reflects the local pathological changes in the urinary bladder, systemic changes, or a combination of both.

Our selection of biomarkers was based on a literature review of previous studies on urine and serum biomarkers [15], as well as recent transcriptomic findings from bladder biopsies in IC/BPS [45, 46]. We expanded our set of measured biomarkers from the classical inflammatory cytokines and chemokines to other biomarkers covering different aspects of disease pathobiology, including factors involved in ECM remodeling, tissue repair, angiogenesis, adhesion and growth factors, and oxidative stress markers.

First, we confirmed elevated urine levels of CCL5, CCL11, CXCL10, BAFF, HGF, 8-izoprostane and oxidized guanine species in IC/BPS patients compared to controls [15–18, 36–38]. Additionally, we identified novel urine biomarkers IL4, MMP1, MMP2, MMP9 and N-cadherin, which are significantly elevated in IC/BPS patients compared to controls.

IL4 is a cytokine released by activated mast cells, which have been reported to be found in increased numbers in the bladders of IC/BPS patients. IL4, in turn, promotes the release of the inflammatory mediators CCL2 and IL6 [47], which have been found to be increased in the urine of patients with IC/BPS in previous studies [37, 38]. In IC/BPS patients, Shen et al. [25] determined that urine levels of IL4 significantly decreased 4 weeks after treatment with extracorporeal shock wave therapy compared to placebo. This study suggested that IL4 might be implicated in the pathophysiology of IC/BPS, however, the study did not include a non-IC/BPS control arm [25].

N-cadherin is a member of the cadherin superfamily that mediates cell-cell interaction in epithelial tissue [48]. Dysregulation of E-, P- and N-cadherins has been associated with tumor invasiveness in various epithelial malignancies, including bladder cancer [49]. Urine N-cadherin levels have been found to be increased in patients with diabetic nephropathy [50]. This is of interest as diabetic nephropathy is associated with increased levels of inflammatory markers and markers of oxidative stress [51, 52], which is also the case for IC/BPS. The elevated levels of N-cadherin found in our study may be related to the inflammatory and oxidative stress process in the bladder.

Secondly, the same biomarkers were also measured in serum samples from the same patients and controls. To date, only four studies have determined serum biomarkers in IC/BPS patients and found elevated levels of CRP, IL1β, IL6, IL8, CXCL10, TNFα, VEGF, nerve growth factor (NGF) and oxidative stress markers [17, 53–55]. In our study, we confirmed higher serum levels of VEGF in IC/BPS compared to controls and lower total antioxidant capacity, while levels of other proteins were either not significant (CRP, IL6, IL8, CXCL10) or not measured (IL1β, TNFα). This discrepancy between different studies might be attributed to the several different commercial kits used for measuring serum proteins, and different routine laboratory techniques or high sensitivity kits used for measuring CRP. Instead, we discovered new serum biomarkers in IC/BPS, including representatives of cytokines (IFNα, IL1RA, BAFF), chemokines (CCL11, CCL27), growth factors (HBEGF, HGF), proteins involved in tissue repair (MMP9, CHI3L1) and the adhesion molecule ICAM1. We also found increased levels of 8-isoprostane, a marker for lipid peroxidation. Significantly modulated serum levels of biomarkers led to better discrimination between IC/BPS patients and controls, compared to significantly modulated urine biomarkers, as revealed by heatmaps and PCA. Based on serum biomarkers, only one patient clustered into the control group. This patient had a very low ICSI score with no Hunner’s lesions almost showing a remission and had a serum biomarker profile more similar to controls than the other IC/BPS patients. Our study showed that the application of a cluster of serum biomarkers could increase the diagnostic value compared to a single biomarker or compared to urine biomarkers and may prove to be a better strategy for future IC/BPS biomarker development.

Four of the biomarkers (CCL11, BAFF, MMP9 and HGF) were elevated in both urine and serum samples from IC/BPS patients, but only CCL11 and BAFF showed a good positive correlation between them. CCL11, also known as eotaxin1, acts as a selective chemoattractant for eosinophils and plays an important role in many eosinophilic inflammatory diseases [38], while BAFF is a major cytokine that belongs to the family of tumor necrosis factors and regulates B cell survival, maturation and differentiation [56]. Elevated urine levels of CCL11 have already been reported in IC/BPS [36–38], while increased expression of BAFF has been found in the bladders of patients with Hunner’s lesions [45]. This is not surprising as the characteristic inflammatory features of IC/BPS with Hunner’s lesions include the accumulation of plasma cells and the frequent expansion of light chain-restricted B cells in the bladder [26]. However, we found no difference in serum or urine levels of BAFF between HIC/BPS and NHIC/BPS, but this may be due to the relatively small sample size, normalization method, and high interindividual variability.

Instead, we found that higher serum levels of MMP9 could discriminate between HIC/BPS and NHIC/BPS with an AUC value of 0.836. Serum levels of MMP9 correlated well with ICSI score, which is also significantly higher in HIC/BPS. MMP9 plays an important role in inflammatory processes as it promotes the release of cytokines, tissue remodeling, angiogenesis and the recruitment of inflammatory cells [57]. MMP9 has so far only been determined in a study that investigated biomarkers of urological chronic pelvic pain syndrome (UCPPS) and also included patients with IC/BPS. Urinary MMP9 levels in male patients with UCPPS were elevated compared to controls and were associated with pain and urinary symptom severity [20]. MMP9 is also involved in the development of neuropathic pain [58]. Kawasaki et al. found increased expression of MMP9 after sciatic nerve ligation in the dorsal root ganglion, while its inhibition attenuated neuropathic pain [59]. Elevated MMP9 levels may therefore reflect an ongoing neurogenic inflammation, which is one of the mechanisms of IC/BPS development and progression [12]. In the context of inflammation and cancer progression, MMP9 is upregulated and may serve to disrupt cell-cell contact through E-cadherin. E-cadherin is a cell–cell adhesion molecule crucial for the maintenance of epithelial structure and tissue homeostasis and is also a direct target of MMP9 [60]. MMP9-induced disruption of the extracellular domain of E-cadherin leads to a reduction in its surface levels and loss of stable cell–cell junctions, apico-basal membrane polarity and epithelial architecture [61]. Downregulated expression of E-cadherin has been reported in IC/BPS bladders [62–64], which may be associated with higher levels of MMP9 determined in urine and serum of IC/BPS in our study. Indeed, a strong correlation between decreased E-cadherin levels and increased MMP9 expression was observed in bladder cancer [61]. The higher MMP9 levels in HIC patients may be related with an Epsteinn-Barr virus (EBV) infection, which was detected in almost 90% of patients with HIC in a previous study [65]. A subsequent study demonstrated the presence of EBV-infected cells (mainly B-lymphocytes) in both Hunner’s lesions and non-inflammed areas of the bladder of HIC patients. They also found upregulation of transcripts encoding EBV-associated inflammatory cytokines, including IL6, CXCL8, IL10, MMP1, TNF, IFNG, CCL3, and CCL4 [66]. Although the expression of MMP9 was not determined, serum levels of MMP9 were found to be higher in patients with oropharyngeal squamous cell carcinoma with EBV infection than in EBV-negative patients [67]. These results and ours suggest that patients with HIC may have higher serum levels of MMP9 due to EBV infection, which warrants further investigation.

Although our study offers several advantages, such as matched urine and serum samples, a high number of biomarkers measured, information on routine laboratory parameters and assessment of clinical symptoms, it also has some limitations. First, the number of patients included was relatively small (n = 12). Second, the included patients and controls were all females, patients were significantly older than controls and a high percentage (58%) of patients with HIC was included, which might have led to a selection bias. Third, urine levels of biomarkers were normalized to the urine creatinine concentration in each sample, which is also a common method in most studies that included IC/BPS patients. However, some studies use normalization to total protein concentration, while other studies report results without normalization. We believe that normalization to urine creatinine is the best option, as creatinine production is usually fairly constant when kidney function, metabolism and muscle mass are stable [68]. Using a comprehensive proteomic analysis of 211 urinary biomarkers in 103 healthy donors, Nolen et al. [69] found that normalizing biomarker measurements based on urinary creatinine levels did not appreciably alter the relative abundance of proteins [69]. Still, the lack of a standardized method for normalization of urine biomarkers leads to difficulties in comparing and validating the results of different studies. 24-hour urine collection would be a better choice to avoid the need for normalization and the influence of circadian rhythm.

Utilizing a comprehensive panel of urine and serum biomarkers, rather than relying on individual markers, holds promise for earlier disease detection in IC/BPS patients. The non-invasive nature of serum and urine biomarkers facilitates frequent testing, potentially enabling timely diagnosis. Given the challenges associated with urine sample collection and analysis, coupled with the compelling findings of this study, we suggest measuring serum biomarkers in IC/BPS patients. Prominent among these biomarkers are CCL11, BAFF, HGF, and MMP9, which exhibit the dual capacity to reflect both local and systemic disease pathology. Their identification not only sheds light on the underlying mechanisms driving IC/BPS development but also presents opportunities for targeted therapeutic interventions.

## Supporting information

S1 Table Correlation between age and significantly modified urine biomarkers in IC/BPS patients. (DOCX)

S2 Table Correlation between age and significantly modified urine biomarkers in controls. (DOCX)

S3 Table Correlation between age and significantly modified serum biomarkers and ICSI score in IC/BPS patients. (DOCX)

S4 Table Correlation between age and significantly modified serum biomarkers in controls. (DOCX)
