# Supplementary material for: Matched serum- and urine-derived biomarkers of interstitial cystitis/bladder pain syndrome
Source: PLoS One. 2024 Dec 31;19(12):e0309815. doi: 10.1371/journal.pone.0309815 (PMC11687793; doi:10.1371/journal.pone.0309815)
Supplement: S1 Table — (DOCX) [file pone.0309815.s001.docx]

|  | Age vs. MMP1 | Age vs. IL4 | Age vs. BAFF | Age vs. HGF | Age vs. CCL11 | Age vs. MMP9 | Age vs. MMP2 | Age vs. CCL5 | Age vs. N-cadherin | Age vs. CXCL10 | Age vs. Oxidized guanine | Age vs. 8-izoprostane |
| --- | --- | --- | --- | --- | --- | --- | --- | --- | --- | --- | --- | --- |
| Spearman r | -0,3789 | -0,2246 | 0,03158 | -0,3368 | -0,5719 | -0,04211 | -0,4912 | -0,3649 | -0,1421 | -0,1860 | -0,2912 | -0,4316 |
| 95% confidence interval | -0,7900 to 0,2672 | -0,7168 to 0,4171 | -0,5656 to 0,6070 | -0,7712 to 0,3114 | -0,8675 to 0,02224 | -0,6137 to 0,5584 | -0,8368 to 0,1341 | -0,7838 to 0,2822 | -0,0926 to -0,38888 | -0,6967 to 0,4498 | -0,7498 to 0,3564 | -0,8125 to 0,2077 |
| P (two-tailed) | 0,2237 | 0,4800 | 0,9253 | 0,2826 | 0,0553 | 0,8994 | 0,1070 | 0,2423 | 0,1276 | 0,5602 | 0,3557 | 0,1619 |
| P value summary | ns | ns | ns | ns | ns | ns | ns | ns | ns | ns | ns | ns |
| Exact or approximate P value | Exact | Exact | Exact | Exact | Exact | Exact | Exact | Exact | Exact | Exact | Exact | Exact |
| Significant? (alpha = 0.05) | No | No | No | No | No | No | No | No | Yes | No | No | No |
| Number of XY Pairs | 12 | 12 | 12 | 12 | 12 | 12 | 12 | 12 | 12 | 12 | 12 | 12 |

**S1 Table. Correlation between age and significantly modified urine biomarkers in IC/BPS patients.**
