# Supplementary material for: Matched serum- and urine-derived biomarkers of interstitial cystitis/bladder pain syndrome
Source: PLoS One. 2024 Dec 31;19(12):e0309815. doi: 10.1371/journal.pone.0309815 (PMC11687793; doi:10.1371/journal.pone.0309815)
Supplement: S2 Table — (DOCX) [file pone.0309815.s002.docx]

**S2 Table. Correlation between age and significantly modified urine biomarkers in controls.**

|  | Age vs. IL4 | Age vs. BAFF | Age vs. HGF | Age vs. CCL11 | Age vs. MMP9 | Age vs. MMP2 | Age vs. N-cadherin | Age vs. CXCL10 | Age vs. Oxidized guanine | Age vs. 8-izoprostane |
| --- | --- | --- | --- | --- | --- | --- | --- | --- | --- | --- |
| Spearman r | 0,1517 | 0,5785 | -0,02116 | -0,07408 | 0,6773 | 0,5080 | 0,4445 | -0,1661 | -0,08113 | 0,3279 |
| 95% confidence interval | -0,4775 to 0,6781 | -0,01242 to 0,8699 | -0,6004 to 0,5727 | -0,6333 to 0,5359 | 0,1503 to 0,9045 | -0,1122 to 0,8433 | -0,1924 to 0,8179 | -0,6860 to 0,4660 | -0,6375 to 0,5308 | 0,06520 to 0,5876 |
| P (two-tailed) | 0,6367 | 0,0521 | 0,9510 | 0,8205 | 0,0182 | 0,0942 | 0,1486 | 0,6026 | 0,8033 | 0,1320 |
| P value summary | ns | ns | ns | ns | * | ns | ns | ns | ns | ns |
| Exact or approximate P value? | Exact | Exact | Exact | Exact | Exact | Exact | Exact | Exact | Exact | Exact |
| Significant? (alpha = 0.05) | No | No | No | No | Yes | No | No | No | No | Yes |
| Number of XY Pairs | 12 | 12 | 12 | 12 | 12 | 12 | 12 | 12 | 12 | 12 |
