# Supplementary material for: Matched serum- and urine-derived biomarkers of interstitial cystitis/bladder pain syndrome
Source: PLoS One. 2024 Dec 31;19(12):e0309815. doi: 10.1371/journal.pone.0309815 (PMC11687793; doi:10.1371/journal.pone.0309815)
Supplement: S3 Table — (DOCX) [file pone.0309815.s003.docx]

**S3 Table. Correlation between age and significantly modified serum biomarkers and ICSI score in IC/BPS patients.**

|  | Age vs. CHI3L1 | Age vs. HBEGF | Age vs. VEGF | Age vs. IL1RA | Age vs. BAFF | Age vs. ICAM1 | Age vs. IFNA | Age vs. HGF | Age vs. CCL27 | Age vs. CCL11 | Age vs. MMP9 | Age vs. Total antiox. | Age vs. 8-izoprostane | Age vs. ICSI |
| --- | --- | --- | --- | --- | --- | --- | --- | --- | --- | --- | --- | --- | --- | --- |
| Spearman r | 0,1193 | 0,3088 | 0,5439 | 0,4421 | 0,1509 | 0,1930 | 0,2655 | -0,08084 | -0,2912 | 0,2842 | -0,3439 | 0,4105 | 0,1754 | 0,2377 |
| 95% CI | -0,5026 to 0,6598 | -0,3394 to 0,7581 | -0,06293 to 0,8571 | -0,1952 to 0,8169 | -0,4782 to 0,6776 | -0,4440 to 0,7004 | -0,3805 to 0,7373 | -0,6373 to 0,5311 | -0,7498 to 0,3564 | -0,3630 to 0,7465 | -0,7743 to 0,3042 | -0,2321 to 0,8037 | -0,4585 to 0,6910 | -0,4056 to 0,7235 |
| P (two-tailed) | 0,7111 | 0,3264 | 0,0706 | 0,1511 | 0,6378 | 0,5451 | 0,3997 | 0,8015 | 0,3557 | 0,3679 | 0,2721 | 0,1850 | 0,5833 | 0,4521 |
| P value summary | ns | ns | ns | ns | ns | ns | ns | ns | ns | ns | ns | ns | ns | ns |
| Number of XY Pairs | 12 | 12 | 12 | 12 | 12 | 12 | 12 | 12 | 12 | 12 | 12 | 12 | 12 | 12 |
