# Supplementary material for: Matched serum- and urine-derived biomarkers of interstitial cystitis/bladder pain syndrome
Source: PLoS One. 2024 Dec 31;19(12):e0309815. doi: 10.1371/journal.pone.0309815 (PMC11687793; doi:10.1371/journal.pone.0309815)
Supplement: S4 Table — (DOCX) [file pone.0309815.s004.docx]

**S4 Table. Correlation between age and significantly modified serum biomarkers in controls.**

|  | Age vs. CHI3L1 | Age vs. HBEGF | Age vs. VEGF | Age vs. IL1RA | Age vs. BAFF | Age vs. ICAM1 | Age vs. IFNA | Age vs. HGF | Age vs. CCL27 | Age vs. CCL11 | Age vs. MMP9 | Age vs. Total antiox. | Age vs. 8-izoprostane |
| --- | --- | --- | --- | --- | --- | --- | --- | --- | --- | --- | --- | --- | --- |
| Spearman r | -0,08113 | -0,3104 | 0,09894 | 0,4304 | 0,4586 | 0,1270 | 0,08797 | 0,1696 | -0,2399 | 0,4238 | 0,1693 | 0,2116 | 0,1979 |
| 95% CI | -0,6375 to 0,5308 | -0,7589 to 0,3378 | -0,5178 to 0,6480 | -0,2092 to 0,8120 | -0,1753 to 0,8237 | -0,4967 to 0,6642 | -0,5259 to 0,6416 | -0,4632 to 0,6879 | -0,7246 to 0,4036 | -0,2169 to 0,8093 | -0,4634 to 0,6877 | -0,4282 to 0,7102 | -0,4399 to 0,7030 |
| P (two-tailed) | 0,8033 | 0,3236 | 0,7584 | 0,1630 | 0,1349 | 0,6930 | 0,7836 | 0,5948 | 0,4497 | 0,1693 | 0,5966 | 0,5062 | 0,5338 |
| P value summary | ns | ns | ns | ns | ns | ns | ns | ns | ns | ns | ns | ns | ns |
| Significant? (alpha = 0.05) | No | No | No | No | No | No | No | No | No | No | No | No | No |
| Number of XY Pairs | 12 | 12 | 12 | 12 | 12 | 12 | 12 | 12 | 12 | 12 | 12 | 12 | 12 |
